# Association of gender with outcomes in critically ill patients

**DOI:** 10.1186/CC11355

**Published:** 2012-05-22

**Authors:** Kamran Mahmood, Kamal Eldeirawi, Momen M Wahidi

**Affiliations:** 1 Department of Medicine, Division of Pulmonary, Allergy and Critical Care Medicine, Duke University Medical Center, DUMC 102356, Durham, NC 27710, USA; 2 Department of Health Systems Science, College of Nursing (M/C 802), University of Illinois at Chicago, 845 S Damen Ave, Room 1054, Chicago, IL 60612, USA

## Abstract

**Introduction:**

The influence of gender on mortality and other outcomes of critically ill patients is not clear. Different studies have been performed in various settings and patient populations often yielding conflicting results. We wanted to assess the relationship of gender and intensive care unit (ICU) outcomes in the patients included in the Acute Physiology and Chronic Health Evaluation (APACHE) IV database (Cerner Corporation, USA).

**Methods:**

We performed a retrospective review of the data available in the APACHE IV database. A total of 261,255 consecutive patients admitted to adult ICUs in United States from 1 January 2004 to 31 December 2008 were included. Readmissions were excluded from the analysis. The primary objective of the study was to assess the relationship of gender with ICU mortality. The secondary objective was to evaluate the association of gender with active therapy, mechanical ventilation, length of stay in the ICU, readmission rate and hospital mortality. The gender-related outcomes for disease subgroups including acute coronary syndrome, coronary artery bypass graft (CABG) surgery, sepsis, trauma and chronic obstructive pulmonary disease (COPD) exacerbation were assessed as well.

**Results:**

ICU mortality was 7.2% for men and 7.9% for women, odds ratio (OR) for death for women was 1.07 (95% confidence interval (CI): 1.04 to 1.1). There was a statistically significant interaction between gender and age. In patients <50 years of age, women had a reduced ICU mortality compared with men, after adjustment for acute physiology score, ethnicity, co-morbid conditions, pre-ICU length of stay, pre-ICU location and hospital teaching status (adjusted OR 0.83, 95% CI: 0.76 to 0.91). But among patients ≥50 years of age, there was no significant difference in ICU mortality between men and women (adjusted OR 1.02, 95% CI: 0.98 to 1.06).

A higher proportion of men received mechanical ventilation, emergent surgery, thrombolytic therapy and CABG surgery. Men had a higher readmission rate and longer length of ICU stay. The adjusted mortality of women compared to men was higher with CABG, while it was lower with COPD exacerbation. There was no significant difference in mortality in acute coronary syndrome, sepsis and trauma.

**Conclusions:**

Among the critically ill patients, women less than 50 years of age had a lower ICU mortality compared to men, while 50 years of age or older women did not have a significant difference compared to men. Women had a higher mortality compared to men after CABG surgery and lower mortality with COPD exacerbation. There was no difference in mortality in acute coronary syndrome, sepsis or trauma.

## Introduction

The association of gender with medical care outcomes is an area of intense interest. However, the studies evaluating this relationship in critically ill patients have reached inconsistent results. Some of these studies are large population-based cohorts, and it may be difficult to apply these conclusions to critically ill patients [1]. Others have evaluated specific disease groups. There are some data to suggest that women have a delay in the diagnostic work up of coronary artery disease and do not receive appropriate and timely cardiac interventions [2-4]. The various outcomes after coronary artery bypass graft (CABG) surgery are worse for women as well [5-7]. The effect of gender on sepsis has varying results in different studies [8-17] while trauma mortality is similar in both men and women [18-21]. Men with chronic obstructive pulmonary disease (COPD) have been shown to have a higher mortality compared to women in some studies [22,23] while others have found similar mortality rates [24]. At this time, however, there is no study on the association of gender with outcomes combining data from a large sample of critically ill patients with a variety of diagnoses from intensive care units (ICU) in the United States (US).

Studies from non-US ICUs have reached different conclusions regarding mortality based on gender, despite men receiving more aggressive care. Gender effects on outcomes in ICU patients in the US may be different from those in European and Canadian centers [25-27], as there are differences in the health care delivery systems. Hence, there is a need to study this issue in critically ill patients in the US.

The Acute Physiology and Chronic Health Evaluation (APACHE) IV database (Cerner Corporation, Kansas City, MO, USA) provides a unique opportunity to answer this question. The data include a large number of ICUs of different sizes, location and teaching status through out the US. It has information on diagnosis, acute physiology scores, age, comorbidities, race, source of patient admission (emergency room, floor, and so on), and time spent at the source. There are data on critical therapeutic interventions and pertinent outcomes, such as length of stay, duration of mechanical ventilation and ICU and hospital mortality [28].

The objective of this study was to evaluate the APACHE IV database for outcomes data regarding gender and diagnosis for a large cohort of critically ill patients. Our hypothesis was that female gender would confer a higher risk for mortality.

## Materials and methods

We conducted a retrospective study of all adults in the APACHE IV database admitted to ICUs in the US between 1 January 2004 and 31 December 2008. Readmissions to the ICU were excluded from the analysis. The permission to access and utilize data was obtained from Cerner Corporation which supplied the data without any patient identifiers.

### Objectives

The primary objective of this study was to evaluate the association of gender with ICU mortality. Secondary objectives included examining the association of gender with the need for active therapy during an ICU stay, the need and duration of mechanical ventilation, the readmission rate, the length of stay in the ICU and hospital mortality. We also assessed the association of gender with ICU mortality in disease subgroups including acute coronary syndrome, CABG surgery, sepsis, trauma and COPD exacerbation.

### Statistical analysis

SAS version 9.1 (SAS Institute, Inc., Cary, NC, USA) was used for all statistical analyses. Univariate analyses were carried out to examine the differences between male and female patients as follows: continuous variables were assessed using Student's t-test and categorical variables were assessed using the Chi-square test. A two-tailed *P*-value of 0.05 or less was considered to indicate statistical significance.

For the analysis of ICU mortality, a multivariate logistic regression model was developed to control for potential confounding variables and to assess for interactions between gender and age. The following variables were included as potential covariates: acute physiology score (APS), age, ethnicity, select chronic health conditions, pre-ICU length of stay, pre-ICU location and hospital teaching status. Ethnicity included African-American, Hispanic and Caucasian while other races were excluded because of the low number of patients. We examined the presence of interaction between age (<50 years versus ≥50 years) and gender in the logistic regression model. We found a statistically significant interaction between the two variables (*P *<0.0001) and, therefore, we conducted the analyses stratified by age group: <50 and ≥50 years of age, controlling for the above covariates. In addition, subsets of admission diagnoses including acute coronary syndrome (including acute myocardial infarction and unstable angina), CABG surgery, sepsis, trauma and COPD exacerbation, were studied individually to assess differences in outcomes based on gender.

Differences between genders in provision of aggressive critical care based on active treatment on the first day in the ICU, and other procedures, such as mechanical ventilation, thrombolytic therapy, pulmonary artery catheter placement, CABG and emergent surgery were also evaluated. Active treatment was defined as one of the thirty-three life saving interventions included in the Therapeutic Intervention Scoring System (TISS) which are unique to or best provided in an intensive care unit [29] and validated in clinical studies [15].

## Results

Our cohort consisted of 261,255 consecutive admissions, of which 144,254 (55.2%) were men and 117,001 (44.8%) were women. Other characteristics of the cohort are shown in Table 1. In bivariate analyses, there were statistically significant differences between men and women on all tested variables but most of these differences were small and most likely due to the large sample size. Of note, there were considerably more men admitted post-operatively (39.7%) than women (32.5%), and men were on average younger (60.6 years) than women (63.1 years).

**Table 1:** Baseline characteristics of the patients admitted to ICU.

Baseline Characteristics	Men Number = 144,254	Women Number = 117,001	*P*-value for difference in means or proportions
AGE (years)	60.6	63.1	<0.0001
RACE (%)			<0.0001
Caucasian	64.5	64.3	
African-American	12.2	14.7	
Hispanic	3.9	5.0	
Asian	1.8	2.0	
American Indian	0.2	0.2	
Other	5.6	5.0	
Missing	11.7	10.2	
COMORBID CONDITIONS (%)			
AIDS	0.7	0.4	<0.0001
Immunosuppression	6.9	7.8	<0.0001
Cirrhosis	2.7	1.8	<0.0001
Hepatic Failure	1.5	1.1	<0.0001
Non-Hodgkins' Lymphoma	0.7	0.6	0.006
Leukemia and Multiple Myeloma	1.1	0.9	<0.0001
Tumor with metastases	3.7	4.7	<0.0001
ADMISSION DIAGNOSIS (%)			<0.0001
Cardiovascular	20.3	20.1	
Sepsis	10.2	11.3	
Pulmonary	7.9	10.3	
Gastrointestinal	6.5	6.1	
Neurological	9.9	11.8	
Surgical	39.7	32.5	
Miscellaneous	5.4	7.8	
LOCATION PRIOR TO ICU ADMISSION (%)			<0.0001
Operating Room	15.5	11.1	
Recovery Room	18.3	19.9	
Emergency Room	36.9	37.5	
Floor	12.1	15.0	
ICU Transfer	2.0	1.8	
Other Hospital	6.1	5.9	
Direct Admission	5.3	4.7	
Step Down Unit	3.7	4.0	
Others	0.1	0.1	
Pre-ICU Length of Stay (hours)	1.6 ± 5.4	1.7 ± 5.2	<0.0001
HOSPITAL TEACHING STATUS (%)			<0.0001
COTH Member	36.2	34.9	
Teaching Hospital, small	35.9	35.8	
Nonteaching Hospital	27.9	29.3	

Continuous variables are compared using Student's t-test and categorical variables are compared using the Chi-square test. A *P*-value of 0.05 or less indicates statistical significance. COTH, Council of Teaching Hospitals.

### ICU mortality

**Figure 1:**
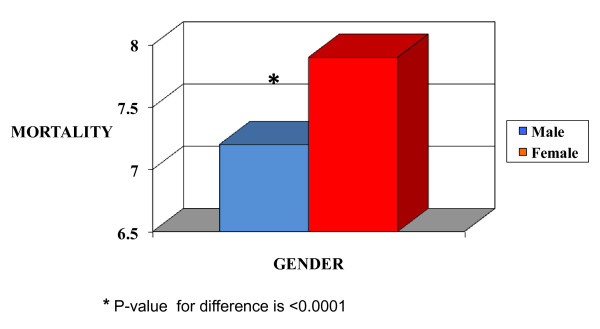
**Crude ICU mortality based on gender**.

**Figure 2:**
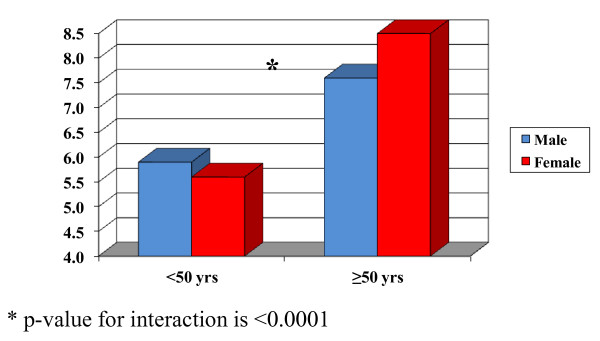
**Crude ICU mortality of gender groups based on age**.

ICU outcomes are shown in Table 2. ICU mortality was 7.2% for men and 7.9% for women (Figure 1), with an odds ratio (OR) of death for women of 1.07 (95% confidence interval [CI]: 1.04 to 1.1, Table 3). However, after adjustment for APS, age, ethnicity, co-morbid conditions, pre-ICU length of stay, pre-ICU location and hospital teaching status, this relationship was not statistically significant (OR 0.98, 95% CI: 0.95 to 1.02). There was a statistically significant interaction between gender and age (Figure 2). In stratified analyses, for patients <50 years of age, women had a lower mortality compared to men with the adjusted OR being 0.83 (95% CI: 0.76 to 0.91). Among patients 50 years of age or older, there was no statistically significant difference in the ICU mortality between men and women, the adjusted OR was 1.02 (95% CI: 0.98 to 1.06), as shown in Table 3.

**Table 2:** ICU outcomes.

ICU Outcomes	Men Number = 144,254	Women Number = 117,001	*P*-value for difference in means or proportions
ICU mortality (%)	7.2	7.9	<0.0001
Hospital mortality (%)	11.8	12.9	<0.0001
Mean APACHE IV	50.3 ± 26	52.6 ± 26	<0.0001
Mean APS	39.1 ± 24	40.3 ± 24	<0.0001
Active treatment day 1 (%)	62.5	60.4	<0.0001
Mechanical ventilation (%)	40.1	37.9	<0.0001
Time on ventilator (days)	1.3 ± 3.8	1.2 ± 3.8	<0.0001
Thrombolytic therapy (%)	1.3	0.7	<0.0001
CABG (%)	6.5	3.1	<0.0001
Emergent surgery (%)	7.2	6.2	<0.0001
Readmission rate (%)	6.2	5.9	0.0005
ICU LOS (days)	4.3 ± 7.4	4.1 ± 6.8	<0.0001
Hospital discharge location (%)			<0.0001
Home	66.7	62.5	
Other Hospital	5.8	5.6	
Hospice	0.3	0.4	
Dead	11.8	12.9	
LTAC/Chronic ventilator Facility	0.03	0.04	
Skilled Nursing Facility	8.4	11.4	
Rehabilitation Facility	3.5	3.4	
Psychiatric Facility	0.03	0.04	
Other	2.5	2.6	

Continuous variables are compared using Student's t-test and categorical variables are compared using the Chi-square test. A *P*-value of 0.05 or less indicates statistical significance. APACHE, Acute Physiology and Chronic Health Evaluation; APS, Acute physiology score; CABG, coronary artery bypass graft; LOS, length of stay; LTAC, long term acute care facility.

**Table 3:** Crude and adjusted odds ratio for mortality in ICU for women compared to men.

ICU Outcomes	Mortality	95% Confidence Interval	*P*-value
Crude mortality	1.07	1.04 to 1.1	<0.0001
Adjusted mortality^a ^	0.98	0.95 to 1.02	0.39
Adjusted mortality for women <50 years^b^	0.83	0.76 to 0.91	<0.0001
Adjusted mortality for women ≥50 years^b^	1.02	0.98 to 1.06	0.46

^a^Adjusted for acute physiology score (APS), age, ethnicity, comorbid conditions, pre-ICU length of stay, pre-ICU location and hospital teaching status. ^b^Adjusted for APS, ethnicity, comorbid conditions, pre-ICU length of stay, pre-ICU location and hospital teaching status. Ethnicity includes African-American, Hispanic and Caucasian while other races were excluded because of the low percentage.

### ICU interventions and outcomes

Mean APACHE IV score on admission to ICU was 50.3 ± 26 for men and 52.6 ± 26 for women (*P*-value <0.0001), while mean APS was 39.1 ± 24 for men versus 40.3 ± 24 (*P*-value <0.0001) as shown in Table 2. There was a statistically significant and higher proportion of men who received active treatment on ICU day 1: mechanical ventilation, pulmonary artery catheters (data not shown), thrombolytic therapy, coronary artery bypass graft and emergent surgery.

More men received mechanical ventilation (40.1% versus 37.9%, *P*-value <0.0001) and they spent more time on the ventilator (1.3 ± 3.8 days versus 1.2 ± 3.8 days, *P*-value <0.0001). There was a higher readmission rate to the ICU for men, 6.2% versus 5.9% for women, OR of readmission for women was 0.95 (95% CI: 0.92 to 0.98). ICU length of stay for men was 4.3 ± 7.4 days and for women was 4.1 ± 6.8 days (*P*-value <0.0001). Hospital mortality of men was 11.8% versus 12.9% for women.

### Subgroup analyses

In additional subgroup analyses (Table 4), among patients who had CABG surgery, women were more likely to die than men (the adjusted OR for mortality: 1.80, 95% CI: 1.28 to 2.54). However, the mortality of women was significantly lower in the COPD exacerbation group (adjusted OR: 0.68, 95% CI 0.53 to 0.87). There was no difference in the adjusted mortality of women with acute coronary syndrome (adjusted OR: 1.25, 95% CI: 0.98 to .59), sepsis (adjusted OR: 1.07, 95% CI: 0.99 to 1.16) and trauma (adjusted OR: 0.89, 95% CI: 0.76 to 1.04).

**Table 4:** Adjusted^a ^odds ratio for mortality in ICU for women compared to men in diagnosis subgroups

Diagnosis Subgroups	Adjusted Mortality	95% Confidence Interval	*P*-value
Acute Coronary Syndrome	1.25	0.98 to 1.59	0.07
CABG Surgery	1.80	1.28 to 2.54	0.0004
Sepsis	1.07	0.99 to 1.16	0.08
Trauma	0.89	0.76 to .04	0.14
COPD exacerbation	0.68	0.53 to 0.87	0.002

^a^Adjusted for acute physiology score (APS), age, ethnicity, pre-ICU length of stay, pre-ICU location and hospital teaching status. Ethnicity includes African-American, Hispanic and Caucasian while other races were excluded because of the low percentage. CABG, coronary artery bypass graft; COPD, chronic obstructive pulmonary disease.

## Discussion

In this analysis of large number of critically ill patients included in the APACHE IV database, the crude ICU mortality was higher for women compared to men. However, there was a significant interaction between gender and age. Since there were differences between men and women in severity of illness, ethnicity, comorbid conditions, pre-ICU length of stay, pre-ICU location and hospital teaching status, we controlled for these covariates. The adjusted mortality of women <50 years was lower compared to men while it was similar in patients ≥50 years of age. In the subgroup analysis, the adjusted mortality of women was higher after CABG and lower for COPD exacerbation. The adjusted mortality was similar for acute coronary syndrome, sepsis and trauma.

Our results are similar to Valentin *et al*. who studied a cohort of patients in Austrian ICUs [25]. They found similar illness adjusted mortality in men and women, despite men receiving an increased level of care. Fowler *et al*. also found a disparity in ICU care in favor of men, with an increased adjusted mortality in older women in Canadian ICUs [26]. In a Belgian retrospective study, Romo *et al*. concluded that women >50 years old had a higher mortality compared to men, while pre-menopausal women failed to show any survival benefit compared to men [27]. Our study is different from these studies as the women younger than 50 years had a lower adjusted ICU mortality, while it was not different in the older women. In our cohort, the APACHE IV and APS were higher in women but more men received active treatment in the ICU, including mechanical ventilation, emergent surgery, thrombolytics and CABG. They stayed longer on the ventilator and in the ICU, and had a higher readmission rate as well.

The age based difference in mortality can be explained on the basis of hormonal theory. Several laboratory studies have shown beneficial effects of estrogen. Estrogen preserves the cardiovascular and immunological function [30,31] and tolerance to severe hypoxia, while 5 α-dihydrotestosterone (DHT) suppresses these functions. Immune responses are more vigorous in women, with greater antibody production and cell mediated immunity after immunization [32]. In an experimental model of *Eschericia coli *lipopolysaccharide administration, females have a greater pro-inflammatory response and norepinephrine sensitivity [33]. Hormonal manipulation in animal models reduces the sepsis related mortality in males. In a hemorrhagic shock model, female rodents have preserved splenocyte function and IL-3 secretion, along with IL-1 secretion by macrophages, in contrast to male rodents [34]. Ovariectomized rodents lose this advantage, which is later regained by administration of 17 β-estradiol [35]. Estrogens reduce the chemotaxis and activation of neutrophils and reduce pulmonary injury following hemorrhagic shock [36]. Administration of estrogen receptor- β agonist preserves gastrointestinal mucosal barrier function and improves outcome in a rodent model of sepsis [37]. 17 β-estradiol-treated males have diminished cardiomyocyte IL-6 production and improved cardiac function [38]. Despite this large accumulation of laboratory evidence on the beneficial effect of estrogens and deleterious effects of testosterone, conclusive evidence in clinical settings is not seen.

Our study showed significantly higher mortality in women after CABG compared to men while mortality in women with an acute coronary event was similar to men in multivariate analysis. Several studies have shown that the outcomes of women after CABG are worse than men, including mortality, duration of mechanical ventilation, length of stay in the ICU and hospital, and some long-term outcomes [5-7]. However, some of the later studies have attributed this to the severity of illness and co-morbidities in women [39-41]. In one study, increased mortality in women after CABG was attributed to infection [42]. Get with the Guidelines-Coronary Artery Database investigators reported no overall difference in mortality between men and women with acute myocardial infarction. However, ST-elevation myocardial infarction mortality was higher in women and there was an underuse of evidence-based therapy and a delay in therapeutic interventions [2]. Similar results have been reported by other researchers [3,4,43].

Women with sepsis had a similar adjusted mortality compared to men in our study. This is in agreement with several studies. In a large cohort of 192,980 patients with severe sepsis, Angus *et al*. concluded that there was no difference in mortality between men and women after adjusting for age, underlying comorbidities and the site of infection [8]. Martin *et al*. in a large epidemiologic study of the US population from 1979 to 2000 found that sepsis was more common in men (annual relative risk 1.28; CI: 1.24 to 1.32). However, they also did not find a difference in mortality in men versus women (22% versus 21.8%) [1]. Several other studies reported similar mortality in both genders [9-11] while others have reported a lower mortality for women [12-14]. In a recent prospective study, Nachtigall and colleagues found a significantly higher mortality for women admitted to the ICU with sepsis while the infection-related care was similar in both the genders [15]. In another study, female patients in the ICU with nosocomial infections had a higher mortality, after controlling for variables including age, severity score, immunocompromised status and admission source [16]. Eachempati and colleagues found that there was a higher mortality in elderly critically ill female surgical patients with sepsis [17]. The difference in outcomes in various studies can perhaps be explained on the basis of different care settings and the innate characteristics of the cohorts. A study has shown that an elevated level of 17 β-estradiol in both genders, increased progesterone in men and elevated testosterone in women is associated with worse survival [44]. However, we do not have any hormonal data available for our cohort.

We found no difference in mortality between the genders admitted with trauma. In a retrospective study by Magnotti and colleagues, there was no difference in mortality after blunt trauma between the genders, but men had a higher morbidity [18]. No difference in mortality was found in several other studies as well [19-21].

The current study showed decreased adjusted mortality in women compared to men admitted with a diagnosis of COPD exacerbation to the ICU. This conclusion is in line with several studies [22,23]. However, Celli and colleagues found that women who participated in the Toward a Revolution in COPD Health (TORCH) study had a lower mortality than men but this difference was not statistically significant after adjustment for important covariates including airflow obstruction and body mass index [24]. Our difference from the TORCH study could be explained on the basis that later excluded patients with significant comorbidities and long-term oxygen therapy.

The current study has several strengths. First, the study has a large sample size of critically ill patients from different demographics and with a wide array of diagnoses. Second, the data used came from a variety of hospitals with different sizes, teaching status, ICU coverage and geographic locations. Thus, the results of the study are generalizable. The weaknesses include a retrospective design that precludes from leading to any definitive conclusions. We cannot rule out other interactions and confounders such as hospital admission time, ICU staffing and so on that may play a role in the observed differences. It is also not clear why men have similar mortality compared to women, despite a trend towards getting more aggressive care. The differences in some of the ICU outcomes including mortality are statistically significant but may be of small magnitude to hold any clinical consequence. It is also possible that these results may be typical for ICUs that use the APACHE IV system and may not be applicable to other institutions. However, this study presents a snapshot of the current critical care practice in the United States.

## Conclusions

This study shows that women younger than 50 years of age had a lower ICU mortality compared to men, after adjustment for covariates. On the other hand, women 50 years of age or older had an adjusted ICU mortality similar to men. There was a higher mortality of women admitted after CABG. However, the mortality of women admitted with COPD exacerbation was less compared to the men. There was no difference in the adjusted mortality between genders with acute coronary syndrome, sepsis and trauma.

## Key messages

• The younger women (<50 years old) admitted to intensive care unit had lower adjusted mortality compared to men, while mortality was similar in older patients.

• Women had higher ICU mortality with CABG surgery and lower ICU mortality with COPD exacerbation compared to men, after adjustment for several clinically important covariates. There was no difference between men and women in adjusted ICU mortality after acute coronary syndrome, sepsis and trauma.

## Abbreviations

APACHE: Acute Physiology and Chronic Health Evaluation; APS: Acute Physiology Score; CABG: coronary artery bypass graft; CI: confidence interval; COPD: chronic obstructive pulmonary disease; DHT: 5 α-dihydrotestosterone; COTH: Council of Teaching Hospitals; LTAC: long term acute care facility.

## Competing interests

The authors declare that they have no competing interests.

## Authors' contributions

KM was responsible for study design, interpretation of data and analysis, and manuscript writing. He takes full responsibility for the integrity of the study. KE was responsible for study design, data analysis, and manuscript revision. MMW was responsible for study design and manuscript revision. All authors read and approved the final manuscript.
